# Therapeutic effect of human umbilical cord mesenchymal stem cells modified by angiotensin-converting enzyme 2 gene on bleomycin-induced lung fibrosis injury

**DOI:** 10.3892/mmr.2014.3025

**Published:** 2014-12-01

**Authors:** FANG MIN, FENGYING GAO, QIAN LI, ZHENWEI LIU

**Affiliations:** 1 Department of Obstetrics, First People’s Hospital Affiliated to Shanghai Jiaotong University, Shanghai 200080, P.R. China; 2 Department of Respiratory Medicine, Shanghai Jiangong Hospital, Shanghai 200083, P.R. China; 3 Department of Pediatrics, First People’s Hospital of Kunshan, Affiliated to Jiangsu University, Kunshan 215300, P.R. China; 4 Department of Respiratory Medicine, First People’s Hospital Affiliated to Shanghai Jiaotong University, Shanghai 200080, P.R. China

**Keywords:** mesenchymal stem cells, angiotensin converting enzyme 2 gene, bleomycin, lung injury, therapeutic effect

## Abstract

The aim of the present study was to evaluate the therapeutic effects of human umbilical cord mesenchymal stem cells (uMSCs) in the presence of angiotensin-converting enzyme 2 gene (ACE2; ACE2-uMSCs) on bleomycin (BLM)-induced lung injury and pulmonary fibrosis in mice. A total of 100 male C57BL/6 mice were divided at random into five groups (n=20) as follows: Control group, BLM group, ACE2 group, uMSC group and ACE2-uMSC group. At 7, 14 and 28 days post-treatment, the following parameters were evaluated in lung tissue: Oxidation indexes [malondialedehyde (MDA), superoxide dismutase (SOD), glutathione (GSH) and oxidized glutathione (GSSG)]; fibrosis factors [tumor necrosis factor (TNF)-α, interferon (IFN)-γ and transforming growth factor (TGF)-β]; inflammatory cytokines [Interleukin (IL)-1, IL-2, IL-6 and IL-10]; ACE2 gene expression; hydroxyproline and collagen type 1 messenger RNA (mRNA) concentration; as well as matrix metalloproteinase (MMPs; 2 and 9) and tissue inhibitor of metalloproteinase (TIMP)1–4 expression. ACE2-uMSC injection following bleomycin pretreatment significantly alleviated lung injury in mice. In addition, treatment with ACE2-uMSCs demonstrated a stronger therapeutic effect than ACE2- or uMSC treatment alone, indicated by decreased expression of MDA, GSSG, TNF-α, IFN-γ, TGF-β, IL-1, IL-2, IL-6, collagen type 1 mRNA, MMPs and TIMPs as well as hydroxyproline concentration, and upregulation of SOD, GSH and ACE2 and IL-10. In conclusion, the results of the present study demonstrated that ACE2 and uMSCs had a synergistic therapeutic effect on bleomycin-induced acute lung injury.

## Introduction

Acute respiratory distress syndrome (ARDS)/acute lung injury (ALI) are severe clinical respiratory failure disorders associated with refractory hypoxemia, which require patients to be kept in intensive care (1). The primary pathogenic risk factors for ARDS/ALI include serious infections or traumatic injury of the respiratory tract, shock, toxic gas inhalation and toxicity (2). The pathogenesis of ARDS/ALI has been well documented by previous studies; however, it still remains a significant problem that results in long-term illness and disability with a high mortality rate of 40–60% (3). The progression of ARDS/ALI consists of three overlapping stages: Exudative, proliferative and fibrotic (4). Detection of pulmonary fibrosis is used as a marker to indicate poor prognosis of ARDS/ALI patients (5). ARDS/ALI therapy currently focuses on repairing alveolar epithelial cells and reducing the severity of pulmonary fibrosis (6,7). Bleomycin-induced lung fibrosis injury was demonstrated to be analogous to ALI in internal medicine diseases, and may therefore be used to study novel therapeutic approaches to the treatment of ALI in animal models.

Previous studies have indicated that mesenchymal stem cells (MSCs) may have potential as a novel therapeutic strategy for the treatment of ALI/ARDS (3). MSCs are multipotent cells derived from adult tissues, which are capable of self-renewal and multi-directional differentiation (into cell types including chondrocytes, osteocytes and adipocytes) (8). Bone marrow mesenchymal stem cells (BMSCs) as well as human umbilical cord mesenchymal stem cells (uMSCs) were reported to differentiate into pulmonary epithelial cells *in vivo* and *in vitro;* these cells were shown to exhibit characteristics specific to lung epithelial cells (9,10). uMSCs may be an ideal and more practical source of MSCs than BMSCs due to their accessibility, pain-free procurement and lack of ethical concerns (11). However, the effectiveness of uMSC treatment for epithelial restitution and reducing fibrosis in ARDS patients requires improvement.

Angiotensin-converting enzyme 2 (ACE2) is a human homologue of ACE (12). ACE2 is primarily involved in the degradation of angiotensin (Ang) II, which results in the formation of Ang 1–7 which opposes the effects of Ang II (13). In brief, ACE2 hydrolyzes Ang II into Ang 1–9, which can be transformed into Ang 1–7 via ACE. Ang 1–7 was reported to block the action of Ang II through the Ang 1–7 G protein-coupled receptor, Mas (14). ACE2 was reported to prevent lung injury resulting from acid inhalation, endotoxin shock and septicemia; by contrast, Ang II promoted fibrosis in mice with bleomycin-induced lung fibrosis injury (15). Current studies into the therapeutic use of ACE2 include increasing its expression using ACE2 adenoviruses, recombinant ACE2 or compounds specific to ARDS/ALI, thereby affording a certain level of organ protection (16–18).

In the present study, ACE2 was transfected into uMSCs via lentiviral vectors. ACE2-modified uMSCs were injected into mice with bleomycin-induced lung injury. The effects of ACE2-uMSCs on lung injury restitution and reduction of fibrosis were subsequently evaluated.

## Materials and methods

### Ethical approval

All human and animal experiments performed throughout the present study were approved by the Human and Animal Research Ethics Committees of the First People’s Hospital affiliated to Shanghai Jiao Tong University, (Shanghai, China).

### Isolation and culture of human uMSCs

Human umbilical cords were obtained, with informed consent, from mothers following normal vaginal delivery at term (n=12). Cords were dissected, washed with D-Hank’s buffer (containing penicillin 100 mg/l and streptomycin 100 U/ml; Invitrogen Life Technologies, Carlsbad, CA, USA), and the blood vessels were removed. The remaining tissue was cut into small pieces (1 mm^2^) and placed in six-well plates at (2.5–5.0)x10^3^/cm^2^ cell density in the presence of Dulbecco’s modified Eagle’s medium supplemented with 10% fetal calf serum (DMEM/F12; Gibco-BRL, Carlsbad, CA, USA). The cell plates were incubated at 37°C in 5% CO_2_ in a humidified incubator. The medium was replaced every three days and images were captured using an inverted microscope (Leitz Labovert FS, Foster City, CA, USA) in order to observe the morphology. The tissue was removed from culture and the fibroblast cells were cultured until they reached 80% confluence in DMEM/F12. Cells were then trypsinized (0.25% trypsin-0.02% EDTA solution, preheated to 37°C; Invitrogen Life Technologies) and re-seeded into culture flasks at 1×10^4^/cm^2^ cell density.

### Determination of cell surface antigen expression

At passage three of the logarithmic phase, cells were harvested using 0.25% trypsin-0.02% EDTA solution. Digestion was terminated by adding fetal calf serum (FCS; Gibco-BRL, Carlsbad, CA, USA). Following centrifugation (1,000 rpm, 10 min), the supernatant was discarded. Cell pellets were washed in phosphate-buffered saline (precooled to 4°C) three times and then suspended evenly. Cells were then incubated with primary monocloncal antibodies, including mouse anti-human CD29, CD34, CD44, CD45, CD86 and CD105 (BD Biosciences, San Jose, CA, USA) at a dilution of 1:50 for 30 min, stained using mouse anti-human fluorescein isothiocyanate (FITC) secondary monoclonal antibodies against CD29-FITC, CD34-FITC, CD44-FITC, CD45-FITC, CD86-FITC and CD105-FITC (BD Biosciences) at a dilution of 1:200 for 30 min, and then analyzed using a flow cytometer (FACSCalibur; Becton Dickinson, Franklin Lakes, NJ, USA).

### Differentiation assays of human uMSCs

At passage five of the logarithmic phase, cells were harvested for preparation of the single-cell suspension and re-seeded into six-well plates at 2×10^3^/ml cell density. Cells were propagated to 60% confluence and then the medium was supplemented with the osteogenic induction medium, containing β-glycerol sulfate (0.5 mmol/l), ascorbic acid (50 mg/l), dexamethasone (0.1 μmol/l; Sigma-Aldrich, St. Louis, MO, USA) and DMEM/F12 (Gibco-BRL), which was replaced every two days. Following three weeks in culture, the cells were subjected to Alizarin Red staining (ARS), Oil red O and immunofluorescent staining to detect the expression of collagen type II (COL II) during the differentiation into osteoblasts, adipocytes and chondrocytes.

### ACE2 gene transfection into uMSCs via lentiviral vectors

The ACE2 complementary DNA (cDNA) sequence was amplified from total RNA with the following primers: Forward: 5′-AAGCTAGCATAGCCAGGTCCTCCTGGCTCCTTC-3′ and reverse: 5′-AAGTCGACCTAAAAGGAAGTCTGAGCATCATCACTG-3′. The amplification of ACE2 was conducted using the following procedure: Denaturation at 98°C for 30 sec, 35 cycles at 98°C for 10 sec, 55°C for 30 sec, 72°C for 90 sec and a final step at 72°C for 10 min, using the Reverse Transcription System. The sequences were then directionally cloned into the lentiviral vector (Invitrogen Life Technologies). The ACE2 lentiviral vector was co-transfected with packaging plasmid (Invitrogen Life Technologies) and envelope plasmid into 293 T cells (Invitrogen Life Technologies).

During the logarithmic phase, uMSCs were trypsinized and adjusted to a suitable cell density using DMEM/F12. The cells were reseeded into 25-ml culture flasks and then cultured in the incubator until cell density reached 2×10^5^/ml. Cells were subsequently transfected via viral vectors. In addition, green fluorescent protein (GFP) was simultaneously run as a control to label uMSCs and its expression was analyzed by fluorescence microscopy (Nikon Eclipse TE200; Nikon Corporation, Tokyo, Japan).

### Establishment of bleomycin-induced lung fibrosis-injury models in mice

A total of 100 C57BL/6 mice (specific pathogen-free grade) were purchased from the Animal Laboratory of FuDan University (Shanghai, China) and randomly divided into five groups (n=20) as follows: Control group (received saline infusion), BLM group (bleomycin pretreatment, received saline infusion), ACE2 group (bleomycin pretreatment, injected with ACE2-cells), uMSC group (bleomycin pretreatment, injected with uMSCs) and ACE2-uMSC group (bleomycin pretreatment, injected with ACE2-uMSCs). Bleomycin (40 mg/ml/kg; Nippon Kayaku Co. Ltd., Tokyo, Japan) was administered intratracheally in order to induce pulmonary fibrosis. Animals were sacrificed following 7, 14 or 28 days of treatment and all parameters were evaluated.

### Pathological observation

Following 7, 14 and 28 days of treatment, five mice from each group were sacrificed. The right lobes of the lung tissue were fixed in 4% paraformaldehyde (Bogoo Biomart, Shanghai, China), embedded in paraffin (Bogoo Biomart), and then stained with hematoxylin-eosin (HE; Bogoo Biomart). Pathological changes of the lung were observed using a light microscope (Nikon, Eclipse E200; Nikon Corporation). Slides were scored for fibrosis using the Ashcroft method (19).

### Examination of oxidative damage

Following 14 days of treatment, five mice from each group were sacrificed and the left lobe of the lung tissue was dissected, weighed and ground into homogenate using saline (1:5). Following centrifugation (3,300 × g, 10 min), the supernatant of the lung homogenate was obtained and frozen until further use. The levels of glutathione (GSH), glutathione disulfide (GSSG) and malondialdehyde (MDA) were determined using the modified fluorescence method. Briefly, the total GSH level was measured using the method of DNTB-GSSG recycling assay, as previously described (20). The GSSG activity was determined by the same method of total GSH assay, once the supernatant was pretreated with solution provided by the manufacturers in order to remove the reduced GSH. The GSH activity was determined by subtracting the GSSG from the total GSH. Results are expressed as molar concentration per mg of protein (nmol/mg). MDA activity was measured by analyzing the reaction of MDA with thiobarbituric acid (TBA), which forms an MDA-TBA2 adduct that absorbs strongly at 535 nm. Results are expressed as (nmol/mg). Superoxide dismutase (SOD) levels were detected using the modified hydroxylamine hydrochloride method as previously reported (21). SOD activity is expressed as unit activity per ml of protein in the sample(U/ml). Commercial assay kits were used to detect GSH, GSSG (GSH and GSSG assay kit), SOD (superoxide dismutase assay kit) and MDA (lipid peroxidation MDA assay kit)levels and were purchased from Westang Co., Ltd, (Shanghai, China). The ratio of GSH/GSSG was then calculated. All procedures were performed according to the manufacturer’s instructions.

### Expression of fibrosis factors and pro-inflammatory factors

Following 14 days of treatment, 30 mg lung tissue was dissected and ground into a homogenate as described above. The supernatant of lung homogenate was used to detect the protein levels of fibrosis factors [tumor necrosis factor (TNF)-α, interferon (IFN)-γ and transforming growth factor (TGF)-β] and inflammatory factors [Interleukin (IL)-1, IL-2, IL-6 and IL-10] using an ELISA kit (ExCellBiology, Inc., Shanghai, China). All procedures were performed according to the manufacturer’s instructions.

### ACE2 expression in lung tissue

Following 7, 14 and 28 days of treatment, 100 mg lung tissue was dissected and ground into powder using liquid nitrogen. The powder was lysed in an Eppendorf tube and agitated using an oscillator every 10 min (total three times), then left to stand for 30 min. Following centrifugation (13,900 × g, 5 min) the supernatant was collected and stored at −80°C for further use for western blot analysis of protein expression.

### Collagen type 1 mRNA expression in lung tissue was measured using quantitative polymerase chain reaction (qPCR)

Total RNA was extracted from lungs of mice following 7, 14 and 28 days of treatment. The Taq-Man probe was obtained from Applied Biosystems (Foster City, CA, USA) for the detection of mouse collagen type 1. Cycling parameters used for qPCR were as follows: Denaturation at 95°C for 7 sec, annealing at 60°C for 7–15 sec and extension at 72°C for 15 sec, for 40 cycles. Data were normalized to β-actin and the expression of the control group was calculated.

### MMP activity and TIMP expression

Following 14 and 28 days of treatment, total RNA was extracted from lung tissue. Matrix metalloproteinase (MMPs; MMP-2 and MMP-9) and tissue inhibitors of matrix metalloproteinase (TIMP; TIMP-1, TIMP-2, TIMP-3 and TIMP-4) mRNA expression in lung tissue was measured using qPCR. Reagents, primers and Taq-Man probes were purchased from Applied Biosystems. Data were normalized to β-actin and the expression of the control group was calculated. The expression of the MMP protein was detected by immunohistochemistry. Lung tisssue were fixed with 4% paraformaldehyde and embedded in paraffin wax, following rinsing and dehydrated. The specimens were sectioned and incubated with mouse anti-MMP-2 and MMP-9 antibody (1:100 dilutions, Santa Cruz Biotechnology, Inc., Dallas, TX, USA). Then the specimen were incubated with peroxidase-conjugated rabbit anti-mouse secondary antibody (Santa Cruz Biotechnology, Inc.) at a dilution of 1:400.

### Statistical analysis

Statistically significant differences between groups were assessed using one-way ANOVA followed by the Bonferroni post-hoc test using SPSS13.0 software for statistical analysis (SPSS Inc., Chicago, IL, USA). Data are presented as the mean ± standard deviation. P<0.05 was considered to indicate a statistically significant difference between values.

## Results

### uMSC phenotype

**Figure 1:**
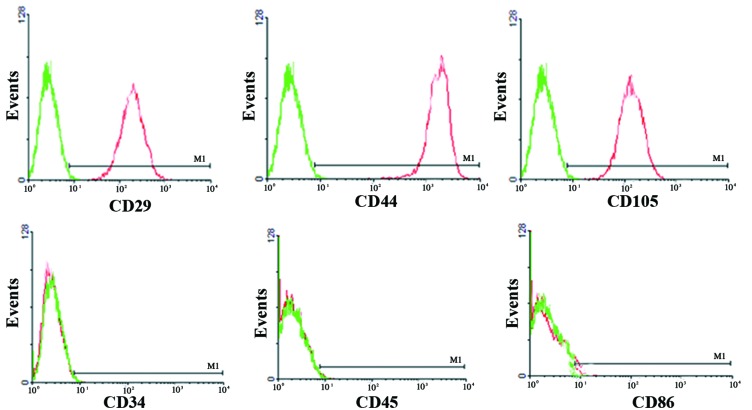
Flow cytometric analysis of cell surface antigens confirms the uMSC phenotype of cells. At passage three of the logarithmic phase, adherent fibroblast-like cells were analyzed for expression of cell surface antigens. Cells expressed the typical mesenchymal pattern of markers: CD29(+), CD44(+), CD105(+), CD34(−), CD45(−), CD86(−). uMSC, human umbilical mesenchymal stem cell.

At passage three of the logarithmic growth phase, adherent fibroblast-like cells were harvested and used to detect the expression of cell surface antigens. Fluorescence activated cell sorting demonstrated that uMSCs expressed the typical mesenchymal pattern of markers, including CD29(+), CD44(+), CD105(+), CD34(−), CD45(−), CD86(−) (Fig. 1).

**Figure 2:**
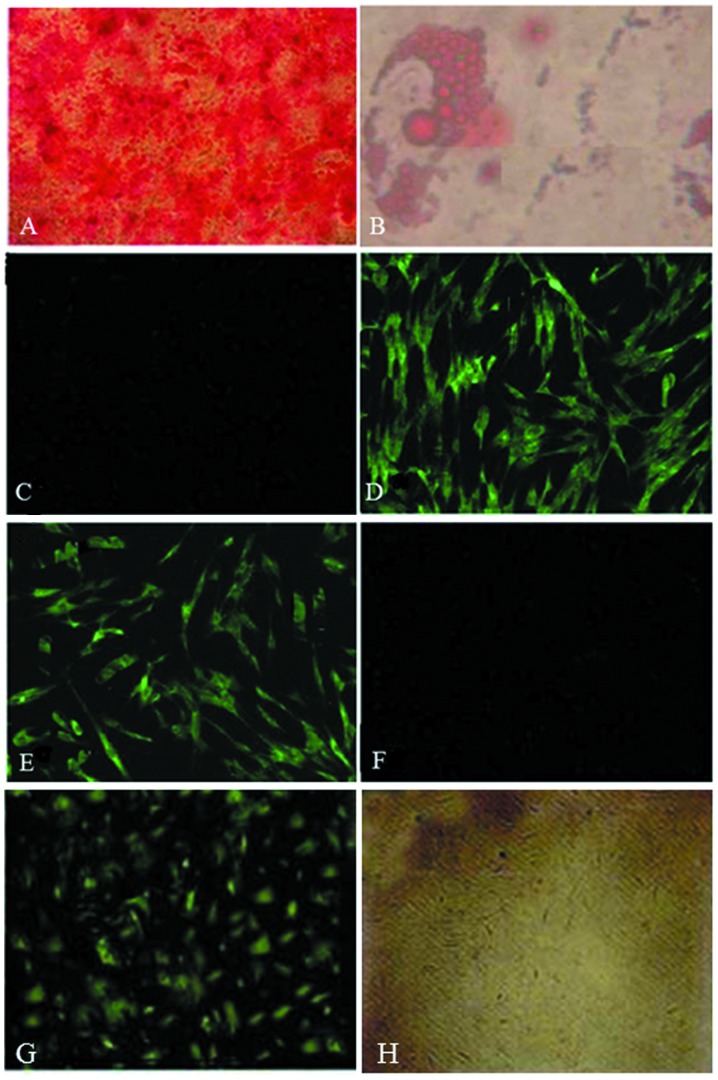
Examination of the differentiation and transfection efficiency of uMSCs. Following transfection with ACE2, uMSCs were stained with (A) Alizarin Red for calcium deposition (orange) to indicate osteoblastic activity and bone formation and (B) oil red O for intracellular lipid droplets (red) to show adipogenic differentiation. Immunofluoresence staining of chondrocytes to reveal expression of collagen type II in the (C) uMSCs only group; (D) ACE2-uMSC group at 14 days and (E) 28 days; and (F) negative control group. Green fluorescent protein-labelled ACE2-uMSCs detected using (G) fluorescence analysis and (H) light microscopy (magnification, ×200). uMSC, human umbilical cord mesenchymal stem cells; ACE2, angiotensin-converting enzyme 2 gene; ACE2-uMSCs, uMSCs transfected with ACE2.

Following 3 weeks in osteogenic induction medium, ARS revealed orange regions that represent calcium deposition and therefore demonstrated bone nodule formation (Fig. 2A). Oil red O staining showed red regions of intracellular lipid droplets, which indicated adipogenic differentiation (Fig. 2B). Immunofluorescent staining of chondrocytes demonstrated a high expression of COL II expression following induction with osteogenic supplements (Fig. 2C–F).

### Efficiency of ACE2 gene transfection into uMSCs via lentiviral vectors

Following 96 h of transfection, GFP detection demonstrated the highest fluorescence intensity (Fig 2G). When the multiplicity of infection (MOI) reached 10, the percentage of GFP-positive cells was >90%. No lesions were detected.

### ACE2-uMSCs significantly alleviate the symptoms of bleomycin-induced lung injury

**Figure 3:**
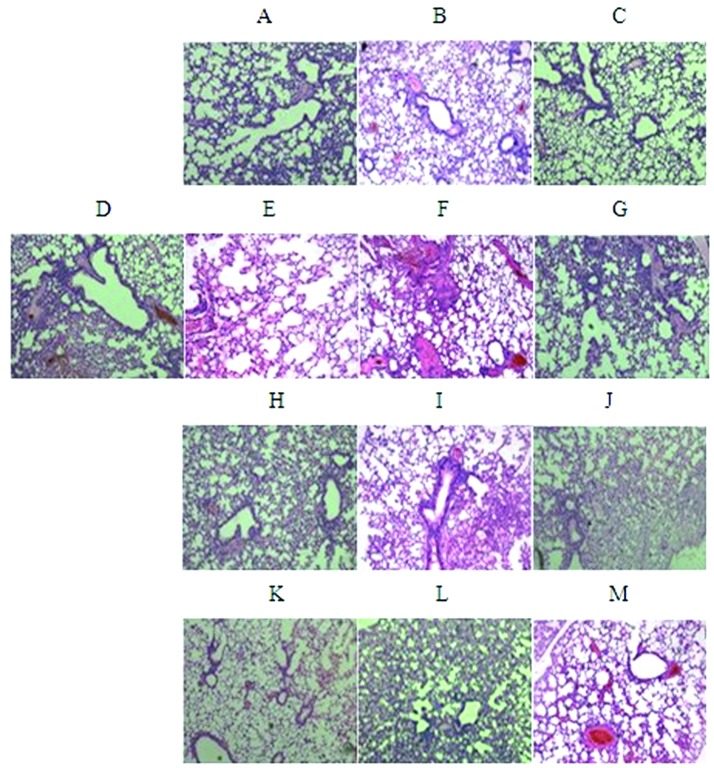
Hematoxylin-eosin staining of lung tissue. Lung tissue sections from each group as follows: ACE2-uMSC group at (A) 7, (B) 14 and (C) 28 days; (D) control group; bleomycin group at (E) 7, (F) 14 and (G) 28 days; ACE2 group at (H) 7, (I) 14 and (J) 28 days; and uMSC group at (K) 7, (L) 14 and (M) 28 days (magnification, ×200). uMSCs, group injected with human umbilical cord mesenchymal stem cells; ACE2, group injected with angiotensin-converting enzyme 2 gene; ACE2-uMSCs, group injected with uMSCs transfected with ACE2; BLM, group with bleomycin-induced lung injury only.

Bleomycin-induced lung fibrosis was established in C57BL/6 mice via pretreatment with bleomycin. In each group, at 7, 14 and 28 days following therapy, HE staining of lung tissue revealed pathological changes, including significant pneumonitis, inflammatory exudates, fibroblastic foci and distortion of the normal architecture of the lung (Fig 3).

The ACE2-uMSC group showed significantly alleviated symptoms (Fig. 2A–C) compared with those of the BLM group (Fig. 3E–G); at day 7, inflammation, hyporrhea and edema were visible but improved; at day 14, stem cells were observed, the structure was less distorted and collagen deposition was reduced; at day 28, the structure of the lung tissues was clear, other symptoms were significantly alleviated and stem cells were not observed. The ACE2-uMSC group also showed significant therapeutic effects compared to those of the ACE2 (Fig. 3H–J) and uMSC (Fig. 3K–M) groups, including reduced inflammation at day 7, reduced collagen deposition at day 14 and clear lung structure at day 28 (Fig. 3A–C). This therefore indicated that factors from uMSCs had significantly contributed to the therapeutic effects of ACE2-uMSCs.

**Figure 4:**
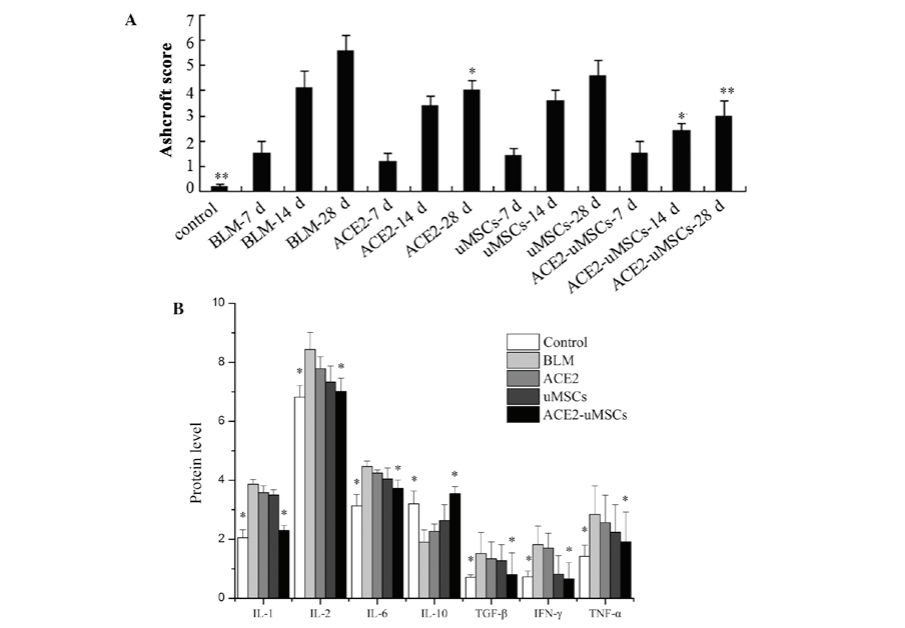
Evaluation of pulmonary fibrosis in the different groups. (A) Ashcroft score of fibrosis for each group at 7, 14 and 28 days following bleomycin-induced lung injury and the control group. (B) Protein expression levels, as determined by ELISA, of profibrotic and proinflammatory factors for each group 14 days following treatment. ^*^P<0.05 and ^**^P<0.01 compared with BLM group. uMSCs, group injected with human umbilical cord mesenchymal stem cells; ACE2, group injected with angiotensin-converting enzyme 2 gene; ACE2-uMSCs, group injected with uMSCs transfected with ACE2; BLM, group with bleomycin-induced lung injury only; IL, interleukin; TGF, transforming growth factor; TNF, tumor necrosis factor; IFN, interferon.

In addition, the Ashcroft score of fibrosis was progressively elevated as lung injury evolved from 7 to 28 days following bleomycin injury (Fig. 4A). The levels of fibrosis at 14 and 28 days following treatment were significantly reduced in the ACE2-uMSC group compared to those of the BLM group.

### ACE2-uMSCs significantly alter oxidation indexes

Following 14 days of treatment, levels of MDA, SOD, GSH and GSSG were detected in the lung tissue of mice from each group (Table I). The ACE2-uMSC group showed significantly reduced MDA and GSSG levels as well as increased levels of SOD and GSH compared to those of the BLM and ACE2 groups (P<0.05). The ACE2-uMSC group also demonstrated significantly reduced MDA levels and increased GSH levels compared to those of the uMSC group (P<0.05); however, the levels of SOD and GSSG were not significantly different. These results therefore indicated that ACE2 alone had no significant effect on preventing oxidative damage and that ACE2 combined with uMSCs had statistically significant effects on the indexes of oxidative damage compared with the BLM and ACE groups, but only altered MDA and GSH levels significantly compared to those in the uMSC group.

### ACE2-uMSCs significantly alter the expression of fibrosis and inflammatory factors

IL-1, IL-2, TNF-α, IFN-γ, TGF-β and IL-6 protein levels were significantly decreased in the ACE2-uMSC group compared with those of the BLM group (P<0.05); whereas, IL-10 was significantly increased (P<0.05) (Fig. 4B). No significant differences in expression of inflammation or fibrosis factors were observed in the ACE2 and uMSC groups compared to those of the BLM group.

### ACE2 expression in lung tissue is significantly increased in the ACE2 and ACE2-uMSC groups

**Figure 5:**
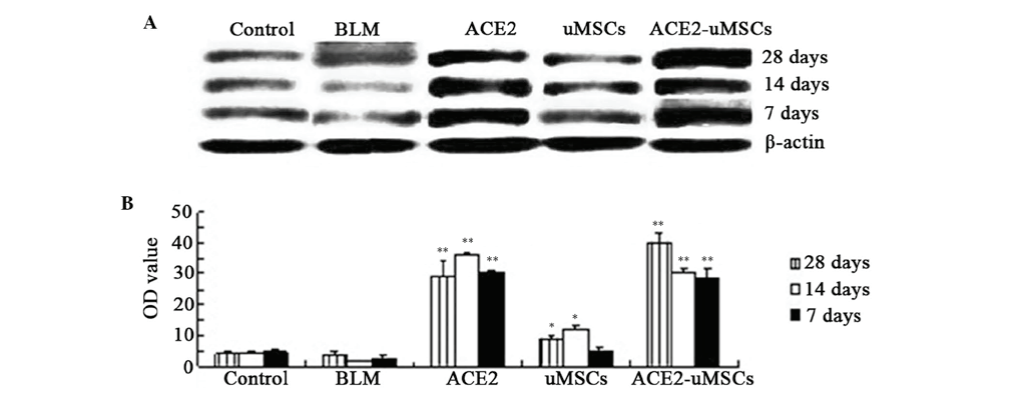
ACE2 expression in lung tissue is significantly increased in the ACE2 and ACE2-uMSC groups. (A) Western blot and (B) densitometric analysis of protein expression levels of ACE2 in lung tissue from each group at 7, 14 and 28 days following injection treatment. β-actin served as control. ^*^P<0.05 and ^**^P<0.01 compared with BLM group. uMSCs, group injected with human umbilical cord mesenchymal stem cells; ACE2, group injected with angiotensin-converting enzyme 2 gene; ACE2-uMSCs, group injected with uMSCs transfected with ACE2; BLM, group with bleomycin-induced lung injury only.

Western blot and densitometric analyses were used to detect the protein expression levels of ACE2 in lung tissue from each group at 7, 14 and 28 days following treatments (Fig. 5). ACE2 expression levels were significantly increased at 7, 14 and 28 days in the ACE2 group as well as the ACE2-uMSC group compared to those of the BLM group (P<0.01) (Fig. 5A and B). Of note, the uMSC group demonstrated significantly increased ACE2 expression levels at 14 and 28 days compared to those of the BLM group (P<0.05). In the ACE2-uMSC group, ACE2 expression levels at each time-point were higher than those of the BLM group and also had a larger increase than those in the ACE2 and uMSC groups at 28 days.

### Collagen deposition is significantly reduced by ACE2 and uMSC treatment individually and in combination

**Figure 6:**
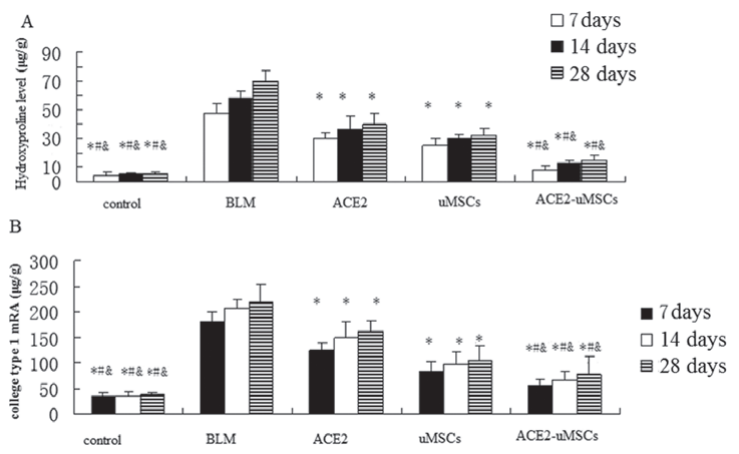
Collagen deposition was significantly reduced by ACE2 and uMSC treatments individually and in combination. Determination of (A) hydroxyproline levels and (B) collagen type 1 messenger RNA in different lung tissue at 7, 14 and 28 days following treatment. ^*^P<0.05 compared with BLM group; ^#^P<0.05 compared with ACE2 group; ^&^P<0.05 compared with uMSC group. uMSCs, group injected with human umbilical cord mesenchymal stem cells; ACE2, group injected with angiotensin-converting enzyme 2 gene; ACE2-uMSCs, group injected with uMSCs transfected with ACE2; BLM, group with bleomycin-induced lung injury only.

Collagen deposition was assessed using a hydroxyproline assay performed on lung tissues at 7, 14 and 28 days following treatment (Fig. 6A). Compared with the BLM, ACE2 and uMSC groups, the administration of ACE2-uMSC decreased expression of hydroxyproline and reduced collagen deposition. In addition, the ACE2 and uMSC groups significantly attenuated collagen deposition compared with the BLM group (P<0.05).

Collagen type 1 mRNA was analyzed using qPCR in order to determine that collagen downregulation was due to reduced synthesis. As shown in Fig. 6B, collagen type 1 mRNA was significantly reduced in mice from the ACE-uMSC group compared to that of the BLM, ACE2 and uMSC groups; furthermore, the ACE2 and uMSC groups significantly attenuated collagen type 1 mRNA expression compared with that of the BLM group. This confirmed that the reduction of lung hydroxyproline levels was due to reduced collagen type 1 synthesis.

### Regulation of MMPs and TIMPs

**Figure 7:**
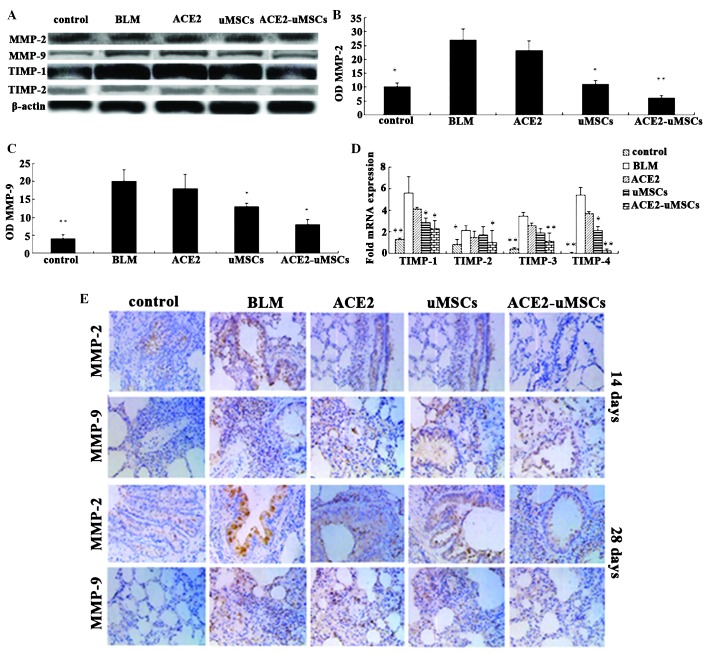
The expression of MMPs (2 and 9) and TIMPs (1–4) in each group at 28 days. (A) Western blot analysis of MMPs (2 and 9) and TIMPs (1 and 2) in each group. Densitometric analysis of (B) MMP-2 and (C) MMP-9 protein expression levels. (D) Quantitative polymerase chain reaction analysis of TIMPs (1–4) mRNA expression. β-actin served as the control. (E) Immunohistochemical analysis of MMP-2 and MMP-9 expression in each group (magnification, ×200). ^*^P<0.05 and ^**^P<0.01 compared with BLM group. MMP, matrix metalloproteinase; TIMP, tissue inhibitor of metalloproteinase; uMSCs, group injected with human umbilical cord mesenchymal stem cells; ACE2, group injected with angiotensin-converting enzyme 2 gene; ACE2-uMSCs, group injected with uMSCs transfected with ACE2; BLM, group with bleomycin-induced lung injury only.

The expression of MMPs (MMP-2 and MMP-9) in whole lung homogenates was determined at 14 and 28 days following treatment (Fig. 7A–C). MMP-2 expression levels were significantly decreased in the ACE2-uMSC and uMSC groups compared to those of the BLM group (P<0.01 and P<0.05, respectively). MMP-9 expression in the five groups was comparable with MMP-2 expression. MMP-9 expression in the ACE2-uMSC and uMSC groups was significantly decreased compared to that of the BLM group (P<0.05).

Immunohistochemical analysis demonstrated different histological expression of MMP-2 and MMP-9 (Fig. 7E). Expression of MMPs was significantly increased in the BLM group compared to that of the control group. Following ACE2-uMSC treatment, MMP expression was significantly decreased compared with that in the BLM group. MMP expression levels at 28 days were higher than those at 14 days.

### The corresponding expression of TIMPs was examined in lung tissues

Transcripts of TIMPs 1–4 were quantified relative to the β-actin control for each group (Fig. 7A and D). uMSC and ACE2-uMSC treatments resulted in reduced expression of TIMP-1 (P<0.05) and TIMP-4 (P<0.05 and P<0.01, respectively) compared with that of the BLM group. Additionally, ACE2-uMSC treatment significantly downregulated the expression of TIMP-2 and TIMP-3 compared to that of the BLM group (P<0.05 and P<0.01, respectively). Western blot analysis of TIMP-1 and TIMP-2 expression levels produced comparable results (Fig. 7A). TIMP-1 and TIMP-2 expression levels were significantly increased in the BLM group compared to those of the control group; by contrast, ACE2-uMSC treatment reduced TIMP-1 and TIMP-2 expression levels significantly.

## Discussion

MSCs, first proposed by Cohnheim (22) in 1867, are a type of adult stem cells from bone marrow and named by Friedenstein *et al* (23). Following delivery of an infant, uMSCs are easily collected and cultured from the umbilical cord (24). To date, the clinical application of uMSCs for the treatment of ARDS/ALI is limited and the therapeutic effect of uMSCs on epithelial restitution and fibrosis reduction of ARDS has not yet been elucidated, to the best of our knowledge.

Previous studies have demonstrated that ACE2 was involved in the pathological processes of numerous lung diseases (25,26). Following the induction of an identical pathogenic environment *in vivo,* ACE2 deletion caused severe ALI in mice, which was reported to be alleviated by injection of ACE2. This therefore indicated that ACE2 acted as a negative regulator of ALI and significantly protected lung tissues (27,28). Microinjection of purified, recombinant ACE2 was reported to reduce collagen deposition, indicating that ACE2 inhibited the formation of pulmonary fibrosis (29). However, to the best of our knowledge, the effect of uMSCs in combination with ACE2 on lung injury and pulmonary fibrosis has not yet been studied. The present study aimed to explore a novel approach for the effective treatment of lung injury and pulmonary fibrosis by transfecting ACE2 into uMSCs.

The results of the present study showed that following bleomycin-induced lung injury, uMSCs were only detected in fibrotic regions at 14 days post-uMSC injection (not detected at 28 days); this therefore indicated that damaged tissues attracted and retained these uMSCs. A previous study reported the transient presence of uMSCs, verifying that the repair of lung injury was consistent with that of other organs and highlighted the importance of trophic factors produced by uMSCs in tissue repair (30). Following ACE2-uMSC injection, expression levels of MDA, GSSG, SOD and GSH were significantly altered compared to those of the BLM group as well as the ACE2 and uMSC groups. The indexes of oxidative damage showed that ACE2-uMSCs effectively reduced the elevated levels of inflammatory oxygen radicals following bleomycin-induced lung injury and demonstrated protective effects on lung tissues. This therefore indicated that ACE2 and uMSCs had a synergistic effect, which was significantly more effective than using ACE2 or uMSCs individually.

To explore the molecular mechanism of the protective ffect of ACE2-uMSC treatment, levels of fibrosis factors (TNF-α, IFN-γ, TGF-β) and inflammatory factors (IL-1, IL-2, IL-6 and IL-10) were determined. In the ACE2 and uMSC groups, the expression levels of inflammatory and fibrosis factors was not significantly different to those of the BLM group. However, previous studies have reported the downregulation of TGF-β by uMSCs, which was associated with reduced collagen deposition (31). The ACE2-uMSC group demonstrated significantly altered expression of all factors examined; therefore, it was suggested that ACE2-uMSC injection inhibited pulmonary fibrosis through downregulation of fibrosis inflammatory factors. Furthermore, the degree of collagen deposition (collagen type 1 mRNA and hydroxyproline) and fibrosis were significantly reduced 28 days following ACE2-uMSC injection compared to that of the BLM group. Individual injections of ACE2 and uMSCs also reduced collagen deposition compared to that of the BLM group; however, injection of ACE2-uMSCs was found to be significantly more effective. The reduction of TGF-β levels and collagen deposition is comparable to that of a previous study, which reported an associated between increased TGF-β levels and the pathogenesis of fibrosis (32).

MMPs and their endogenous inhibitors (TIMPs) are primarily responsible for the degradation of extracellular matrix proteins such as collagen (33). MMP-2 and MMP-9 were downregulated following ACE2-uMSC treatment in bleomycin-injured mice, contrary to previous studies (31). This difference may be due to the source of MSCs and the mouse strain (C57BL/6) used. In addition, Ortiz *et al* (25) reported the downregulation of MMP-2 by murine BMSCs following bleomycin-induced fibrosis. In the present study MMP-2 expression levels were significantly decreased in the ACE2-uMSC group, compared to those of the BLM and uMSC groups. Previous studies have indicated MSCs may reduce pulmonary fibrosis via inhibition of MMP expression (34,35). In addition, ACE2-uMSCs significantly reduced the expression of TIMP1–4, which was concurrent with the results reported by a previous study (36). In addition, ACE2-uMSCs were found to be more effective in the reduction of TIMP expression than ACE2 or uMSCs alone. In the ACE2-uMSC group, the MMP-2/TIMP-2 ratio was relatively balanced, which may have promoted the protection of injury-induced pulmonary fibrosis.

In conclusion, injection of ACE2-uMSC demonstrated significantly more effective results in the treatment of bleomycin-induced pulmonary fibrosis *in vivo* compared to those of the ACE2 and uMSC treatments alone. The results of the present study therefore suggested that the synergistic effect of ACE2 and uMSCs may be used as a promising novel treatment for lung injury.

## Figures and Tables

**Table I:** MDA, SOD, GSH and GSSG levels of lung tissues after 14 days of different treatments.

Group	MDA (nmol/mg)	SOD (U/ml)	GSH nmol/mg)	GSSG (nmol/mg)	GSH/GSSG
Control	3.1±0.05^a^,^b^,^c^	30.1±2.36^a^,^b^,^c^	664±20.55^a^,^b^,^c^	309±10.22^a^,^b^,^c^	2.14±0.04^a^,^b^,^c^
BLM	8.3±0.11	16.9±1.43	382±12.32	523±18.37	0.73±0.02
ACE2	7.5±0.09	19.3±1.33	412±15.87	501±17.79	0.82±0.02
uMSC	7.1±0.08	21.4±1.92	463±17.76^a^.^b^	458±16.93^a^	1.01±0.03^a^
ACE2-uMSC	5.7±0.06^a^,^b^,^c^	25.8±2.05^a^,^b^	577±19.06^a^,^b^,^c^	401±11.46^a^,^b^	1.43±0.04^a^,^b^

a P<0.05 compared to BLM group;

b P<0.05 compared to ACE2 group;

c P<0.05 compared to uMSC group.

MDA, malondialdehyde; SOD, superoxide dismutase; GSH, glutathione; GSSG, glutathione disulfide; uMSCs, group injected with human umbilical cord mesenchymal stem cells; ACE2, group injected with angiotensin-converting enzyme 2 gene; ACE2-uMSCs, group injected with uMSCs transfected with ACE2; BLM, group with bleomycin-induced lung injury only. Data are presented as the mean ± standard deviation.
